# The Effect of *Elaeagnus angustifolia L.* Leaf Extract on *Encephalitozoon intestinalis*, *Acanthamoeba castellanii*, and *Leishmania major*

**DOI:** 10.1007/s11686-026-01246-9

**Published:** 2026-03-09

**Authors:** Ülfet Çetinkaya, Gülay Sezer, Ayşe Gül Bakkal Zorlu, Türkan Mutlu Yar, Melike Öztürk, Gülşah Avcı, Ülkü Karaman

**Affiliations:** 1 Health Vocational High School, Erciyes University, 38039 Talas, Kayseri Turkey; 2 Genome and Stem Cell Center (GENKOK), Erciyes University, Kayseri, Turkey; 3 Faculty of Medicine, Department of Pharmacology, Erciyes University, Kayseri, Turkey; 4 ERFARMA Drug Application and Research Center, Erciyes University, Kayseri, Turkey; 5 Graduate School of Health Science, Erciyes University, Kayseri, Turkey; 6 Faculty of Medicine, Department of Parasitology, Ordu University, Ordu, Turkey; 7 Gevher Nesibe Genome and Stem Cell Institute, Erciyes University, Kayseri, Turkey

**Keywords:** Encephalitozoon intestinalis, Acanthamoeba castellanii, Leishmania major, Elaeagnus angustifolia L., Traditional medicine

## Abstract

**Purpose:**

Parasitic diseases are a major global health concern. Treating these diseases presents many challenges. *Elaeagnus angustifolia L*. (EA) is renowned for its anti-oxidant, anti-inflammatory, and anti-microbial properties, and various parts of the plant are used to treat a variety of ailments. This study aims to evaluate the in vitro activity of the EA leaves against *Encephalitozoon intestinalis* (*E. intestinalis*), *Acanthamoeba castellanii* (*A. castellanii*), and *Leishmania major* (*L. major*) at different concentrations and incubation times.

**Methods:**

Spore load was measured by real-time PCR using an infection model in human kidney epithelial (HEK) 293 cells for *E. intestinalis*. The viability of *A. castellanii* trophozoites and cysts, and *L. major* promastigotes was determined by trypan blue staining and hemocytometry.

**Results:**

*Elaeagnus angustifolia L.* leaf extract significantly reduced the spore DNA load in *E. intestinalis* infections at a concentration of 5 µg/mL, but was ineffective at lower concentrations. The extract decreased the viability of *A. castellanii* trophozoites and cysts, as well as *L. major* promastigotes, at varying rates depending on the time and dose. It was particularly effective against *A. castellanii* cysts at low doses.

**Conclusion:**

The biological activity of the plant extract obtained from the leaves of *Elaeagnus angustifolia L.* against three different parasites suggests that it could be used as a promising alternative in the treatment of parasitic infections.

## Introduction

Parasitic diseases are a major global public health problem, affecting millions of people, particularly in tropical and subtropical regions, leading to serious morbidity and mortality. These infections also cause significant economic losses [1]. One of the most critical issues in controlling these diseases is the difficulty of treatment and the increasing prevalence of drug resistance. For example, microsporidian infections are difficult to treat, and existing drugs are only partially effective [2]. Similarly, no single preparation is effective against all Acanthamoeba isolates [3]. Likewise, resistance to antimony compounds in Leishmania species reduces the success of treatment [4]. The development of resistance is influenced by various biological processes, such as mutations in drug targets, overexpression of drug transporters, and metabolic bypass mechanisms [5]. As this complex resistance structure severely limits the effectiveness of existing treatments, innovative, more effective alternative treatment options must be developed.

Throughout history, plant extracts have been used in traditional medicine, and the development of modern pharmacology has led to a re-evaluation of their therapeutic potential. Plants naturally contain a variety of bioactive molecules, including alkaloids, flavonoids, terpenoids, phenolic compounds, and glycosides. These molecules can exert a wide range of pharmacological effects due to their antioxidant, anti-inflammatory, anti-microbial, anti-viral, and immunomodulatory properties [6]. The presence of diverse bioactive compounds allows these natural preparations to act on multiple targets simultaneously, giving them a distinct advantage over single-target synthetic drugs [7]. Furthermore, the low toxicity and better tolerability profiles of many plant extracts in long-term use have raised curiosity in their use in complementary and alternative medicines [8].

*Elaeagnus angustifolia L*. (EA), a deciduous shrub that can grow up to seven meters tall, has been used in traditional Middle Eastern medicine for centuries [9, 10]. It has pointed, short-stemmed, long-elliptical leaves and small, reddish-brown fruits that are 1.5–2.0 cm long and also elliptical in shape. Due to its resistance to high salinity, severe drought, and soil alkalinity, it can grow in very arid areas [11]. It occurs naturally in Europe, Türkiye, the Caucasus, Syria, Iran, Afghanistan, and Pakistan, and is widely distributed throughout Türkiye [9]. Various parts of EA, which are a rich source of vitamins, protein, calcium, magnesium, potassium, and iron, are consumed fresh or dried [12]. EA is known for its anti-oxidant, anti-inflammatory, anti-microbial, and anti-cancer properties, and various parts of the plant are used to treat many diseases [13, 14]. EA leaves are boiled and consumed to lower blood sugar, while leaf extracts are used as diuretics and antipyretics [11, 12]. Therefore, it is thought that it may have anti-parasitic effects against *Encephalitozoon intestinalis* (*E. intestinalis*), *Acanthamoeba castellanii (A. castellanii)*, and *Leishmania major* (*L. major)*, which are challenging to treat. No studies demonstrating the anti-parasitic effects of this plant were found in the available sources.

This study aims to evaluate the in vitro activity of the EA leaves against *E. intestinalis*, *A. castellanii*, and *L. major* and thereby explore the potential of natural products in the treatment of parasitic diseases.

## Materials and Methods

### Plant Material and Preparation of the Extract

The leaves of the EA plant were collected in September 2024 during the fruiting season from Hamurcu village in the İncesu district of Kayseri, Türkiye, at an altitude of 1675 m. The plant material was identified by Prof. Dr. Cem Vural, a faculty member in the Biology Department at Erciyes University’s Faculty of Science, using international identification methods. The leaves were washed in distilled water to remove contamination and then dried passively in a clean, dust-free, and shaded area in preparation for extraction.

Extraction was performed by macerating 20 g of the plant material in 400 mL of 80% methanol for 24 h. The resulting extracts were filtered through filter paper and evaporated at 40 °C to remove the solvent. The extracts were then lyophilized. The extracts were stored at + 4 °C until analyzed [15]. % yield of extract was 21.04%.

### Total Phenolic and Total Flavonoid Content

The total phenolic content was determined using the Folin-Ciocalteu analysis, a spectrophotometric method. The plant extract and Folin-Ciocalteu reagent (diluted 1:9) were mixed, and then a 1% sodium carbonate (Na_2_CO_3_) solution was added. The mixture was left at room temperature for two hours before the absorbance was measured at 765 nm. The same procedure was followed for gallic acid, which was used as the standard. Total phenolic content was expressed as gallic acid equivalents (mg GAE/g) [15, 16].

The aluminium chloride (AlCl_3_) method was used to determine the total flavonoid content. The plant extract was mixed with a methanolic solution of 2% AlCl_3_. After the mixture was left to stand for 10 min, the absorbance of the mixture was measured against the blank at 415 nm. The same procedure was followed for the standard routine. The total flavonoid content of the extract was expressed as rutin equivalents (mg RE/g) [15, 17].

### Biological Activities Evaluation

The 2,2-diphenyl-1-picrylhydrazyl (DPPH) assay was selected for evaluating biological activities in this study due to its simplicity, rapid execution and widespread use in assessing the antioxidant capacity of plant extracts, particularly those rich in phenolic and flavonoid compounds. Furthermore, the DPPH method is suitable for measuring hydrogen-donating antioxidants with moderate polarity, consistent with the expected chemical characteristics of ethanolic leaf extracts [18]. For the DPPH test, a 0.004% methanolic DPPH solution was prepared. This was then mixed with the plant extract and allowed to stand at room temperature for 30 min before the absorbance was measured at 517 nm. Trolox was used as a standard, and the results were expressed as Trolox equivalents (mg TE/g) [15, 19].

### Cytotoxic Potential

The effect of the extract on host cell viability was analyzed using the 3-(4,5-Dimethylthiazol-2-yl)-2,5-diphenyltetrazolium bromide (MTT) assay. HEK293 cells were selected for this assay because they were also used as the host cell model in the *E. intestinalis* infection experiments. First, HEK293 cells (1 × 10^4^ cells in 100 µL) were plated in wells of 96-well plates and incubated at 37 °C in 5% CO_2_ overnight. A total of 10 mg of the plant extract was weighed, dissolved in 10 ml of culture medium and used to make an initial stock solution (1 mg/ml). This solution was sterilised by filtration, then diluted 1:1 with culture medium to produce a working stock solution at a concentration of 500 µg/mL. This working stock solution was then used to prepare the remaining test concentrations by dilution with the culture medium, as detailed below: 500 µL of working stock mixed with 500 µL of medium to obtain 250 µg/mL; 250 µL of working stock mixed with 750 µL of medium to obtain 125 µg/mL; 150 µL mixed with 850 µL of medium to obtain 75 µg/mL; 100 µL mixed with 900 µL of medium to obtain 50 µg/mL; 50 µL mixed with 950 µL of medium to obtain 25 µg/mL; 25 µL mixed with 975 µL of medium to obtain 12.5 µg/mL; 10 µL mixed with 990 µL of medium to obtain 5 µg/mL. All dilutions were freshly prepared prior to each experiment. The media were then replaced with growth media containing various concentrations of the extract (500, 250, 125, 75, 50, 25, 12.5, and 5 µg/mL) and incubated at 37 °C in 5% CO_2_. At the 48th hour, the media were replaced with growth media containing the same concentrations. After 48 h, at the 96th h, 10 µL of MTT solution was added to each well and incubated for 4 hours at 37 °C. Then, 100 µL of dimethyl sulfoxide (DMSO) was added to solubilize the purple formazan crystals, and the cells were incubated for 20 min at 37 °C. Absorbance was measured at 560 nm. The data were represented as a percentage of cell viability relative to the control (HEK 293 cells cultured in medium without plant extract) cells as previously described [20].

#### Effects on *Encephalitozoon intestinalis*

The *E. intestinalis* reference strain was obtained from the American Type Culture Collection (ATCC 50506). The parasites were cultured in HEK293 cells, as previously reported [21]. To assess the microsporicidal effects of EA leaves extract on *E. intestinalis*, 1.3 × 10^4^ HEK293 cells were seeded in wells of 12-well tissue culture plates in 1000 µL growth medium and incubated for 24 h at 37 °C in 5% CO_2_. After the removal of non-adherent cells, 4.6 × 10^6^ spores were added to each well and incubated for an additional 24 h. The next day, non-adherent spores were removed by washing with DPBS. Fresh medium containing various concentrations of extract (5, 2.5, and 1 µg/ml) was added to the wells and incubated at 37 °C in 5% CO_2_. These concentrations were selected based on cytotoxicity assays, which showed that 5 µg/mL was non-cytotoxic to HEK293 cells, while lower concentrations were included to evaluate dose-dependent effects. A group infected with *E. intestinalis* but not treated with the extract was used as a control. This was done to evaluate the effectiveness of the treatment. The media was replaced every 48 h, and a total of five doses of extract were administered. Ten days after the infection, spores became detectable in the medium, and the experiment was terminated. To quantify parasite density, all media and host cells were removed from the cell culture plate with trypsin and collected in a tube. The tubes were then centrifuged at 4000×g for 10 min, and the supernatant was removed. The pellet was used for DNA isolation [20].

DNA was prepared using the GeneAll^®^ Exgene Cell SV Mini Kit (GeneAll Biotechnology, Seoul, South Korea) according to the manufacturer’s recommendation. Real-time PCR reactions and determination of spore loads in the groups were performed as previously described. Spore load was expressed as based on parasites DNA, using a standard curve generated from serial dilutions of a plasmid encoding the *E. intestinalis* 16 S SSU rRNA region. All experiments were performed independently three times. Statistical analyses were conducted by comparing treated groups with the infected, untreated control group [22]. Differences were considered statistically significant at *P* < 0.05, while non-significant differences were indicated as NS.

#### Effects on *Acanthamoeba castellanii*

*Acanthamoeba castellanii* genotype T5, molecularly confirmed by 18 S rRNA gene analysis, was used in this study and was obtained from provided by Prof. Dr. Zübeyda Akın Polat (Sivas Cumhuriyet University). Cyst and trophozoite cultures were performed as previously described [23–25]. To test the effect of the extract on trophozoites, 5 × 10⁴ trophozoites were seeded into wells of 96-well tissue culture plates in 100 µL of PYG medium, composed of peptone (20 g/L), yeast extract (20 g/L), glucose (18 g/L), NaCl (120 mg/L), MgCl₂·6 H₂O (3 mg/L), Na₂HPO₄ (142 mg/L), KH₂PO₄ (136 mg/L), CaCl₂ (3 mg/L), and FeSO₄·7 H₂O (3 mg/L), prepared in distilled water, autoclaved at 121 °C for 15 min, and adjusted to pH 5.6 [25]. A PYG medium containing the following final concentrations of extract was added to the wells and incubated at 30 °C: 5 µg/mL, 12.5 µg/mL, 25 µg/mL, 50 µg/mL, 125 µg/mL, 250 µg/mL, 500 µg/mL, and 1000 µg/mL. A group not treated with the extract was used as a control. This was done to evaluate the effectiveness of the treatment.

To test the effect of the extract on cysts, the trophozoite culture was centrifuged at 500 × g for 10 min. The pellet was resuspended in cyst culture medium composed of NaCl (5.5519 g/L), KCl (0.3728 g/L), MgSO₄ (0.963 g/L), NaHCO₃ (0.084 g/L), CaCl₂ (0.044 g/L), and Tris-HCl (3.152 g/L), prepared in one liter of distilled water, autoclaved at 121 °C for 15 min, and adjusted to pH 9 [24, 25]. The suspension was then incubated at 25 °C in a controlled incubator for 6 days. The culture was examined under a light microscope, and complete transformation of trophozoites into cyst forms was observed. 3 × 10^4^ cysts were seeded in wells of 96-well tissue culture plates in 100 µL cyst medium [25]. A medium containing the following final concentrations of extract was added to the wells and incubated: 5 µg/mL, 12.5 µg/mL, 25 µg/mL, 50 µg/mL, 125 µg/mL, 250 µg/mL, 500 µg/mL, and 1000 µg/mL. A group not treated with the extract was used as a control. This was done to evaluate the effectiveness of the treatment.

Trophozoites and cysts were stained with trypan blue at 24, 48, and 72 h, and the viability was determined.

#### Effects on *Leishmania major* promastigotes

The *Leishmania major* strain (MHOM/TR/2013/MANISAPB145) was obtained from the parasite bank of Celal Bayar University. Promastigotes were primarily cultured on Novy–MacNeal–Nicolle (NNN) medium. After entering the logarithmic growth phase, it was transferred to Roswell Park Memorial Institute (RPMI)-1640 medium supplemented with 15% FBS [26].

To test the effect of the extract on promastigotes, 1.2 × 10^5^ promastigotes were seeded in wells of 96-well tissue culture plates in 100 µL RPMI-1640 medium. A medium containing the following final concentrations of extract was added to the wells and incubated at 25 °C for 24, 48, and 72 h: 5 µg/mL, 12.5 µg/mL, 25 µg/mL, 50 µg/mL, 125 µg/mL, 250 µg/mL, 500 µg/mL, and 1000 µg/mL. A group not treated with the extract was used as a control. This was done to evaluate the effectiveness of the treatment. At the end, the direct counting method by hemocytometer chamber was performed to evaluate the anti-leishmanial effects [27, 28].

### Statistical Analysis

Data are expressed as the mean ± standard error of the mean (SEM). All statistical analyses were performed using the statistical package for the social sciences (SPSS) version 22.0 software package (IBM, Chicago, IL). The distribution of the data was evaluated using the Shapiro–Wilk tests. Where the data were normally distributed, comparisons between groups were made using one-way analysis of variance (ANOVA), followed by a Tukey’s post hoc test. For non-normal distributions, variables were compared using a Kruskal-Wallis analysis, followed by a Mann–Whitney U analysis for pairwise comparisons. Values for *p* < 0.05 were considered statistically significant.

## Results

### Total Bioactive Compounds (total Phenolic and Flavonoid content) and Total Anti-oxidant Activity

The total phenolic content of the extract obtained from the plant’s leaves was found to be 157.47 mg GAE/g. The total flavonoid content was 32.34 mg RE/g, while the DPPH radical scavenging activity was 279.32 mg TE/g.

### Cytotoxic Potential

**Fig. 1:**
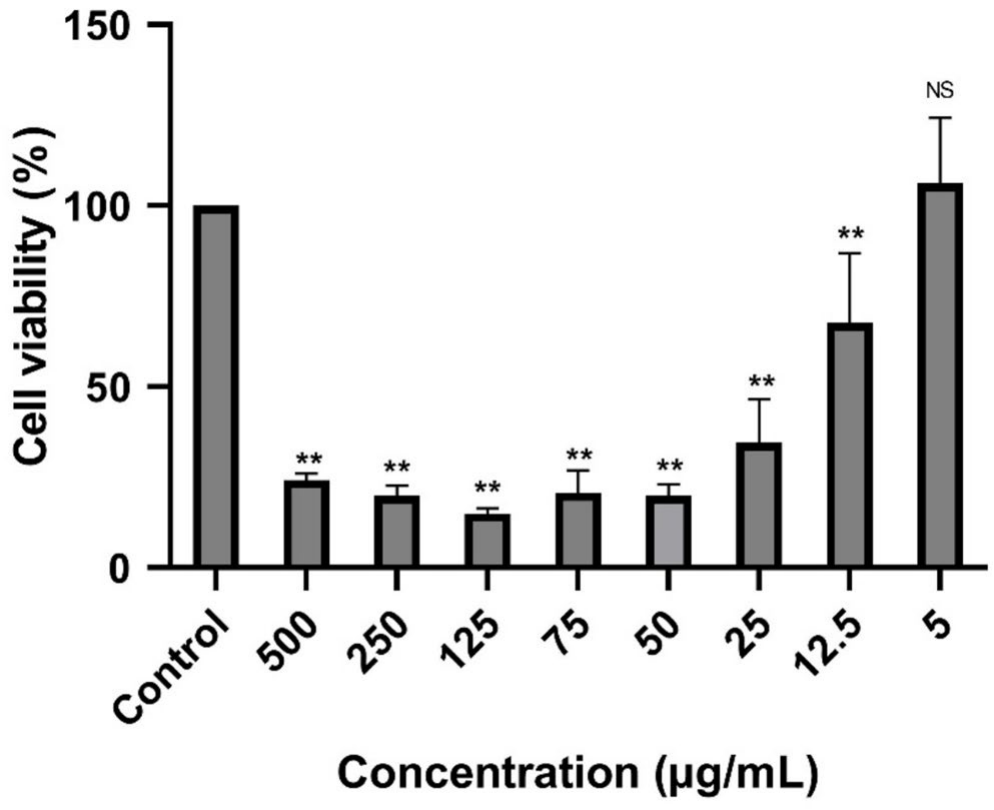
% Cell viability of host cells treated with various concentrations of the extract (500, 250, 125, 75, 50, 25, 12.5, and 5 µg/mL) and control (HEK 293 cells cultured in medium without plant extract). Bars represent the mean ± SD of three independent experiments. ∗*P* < 0.05, ∗∗*P* < 0.005 represents statistical significance vs. control

According to the results of the cytotoxicity test, the 5 µg/mL concentration of the extract had no toxic effect on the host cells (*p* > 0.05). However, the difference in viability rate at 12.5 µg/mL was statistically significant compared to the control group (*p* < 0.005); therefore, these concentrations were considered cytotoxic (Fig. 1). Consequently, we analyzed the anti-microsporicidal efficacy of the three non-toxic concentrations (5 µg/mL, 2.5 µg/mL, and 1 µg/mL) of extract.

### Anti-microsporidial Activity

**Fig. 2:**
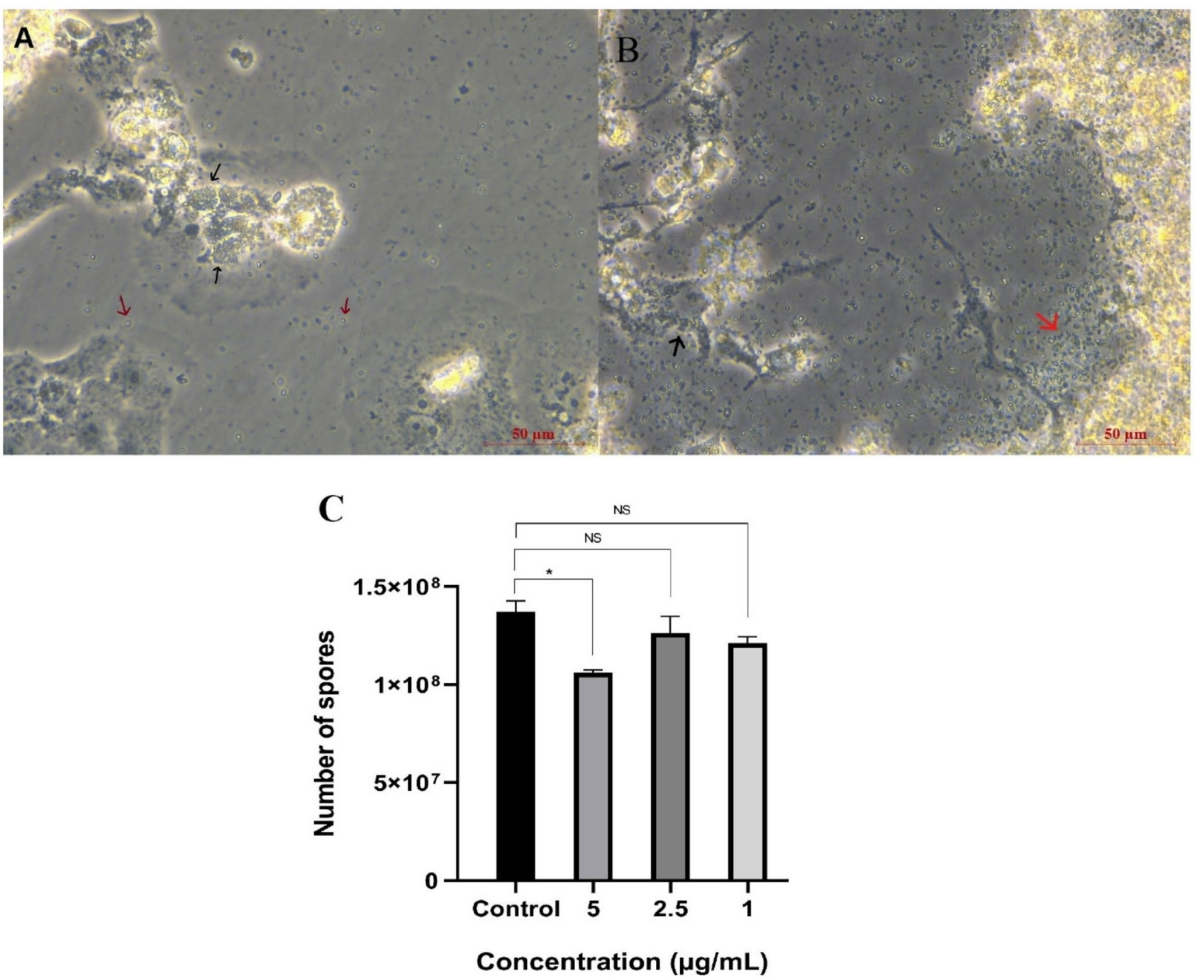
The microsporicidal effects of EA leaves extract. **A**, **B** Images showing *E. intestinalis* spores (red arrows) and foci of infected cells (black arrows) in culture medium after 10 days of incubation. **C** Spore load based on parasite DNA determined by qRT-PCR analysis of the in vitro effects of plant extract concentrations (5 µg/mL, 2.5 µg/mL, and 1 µg/mL) on *E. intestinalis* after 10 days of incubation. Data represent the mean ± SEM, and all experiments were performed in triplicate. NS, not significant; *P* < 0.05 vs. control (*E. intestinalis*–infected, untreated group). Quantification of spore load was performed using a PCR-based approach. The dilution series prepared from the plasmid encoding the *E. intestinalis* 16 S SSU rRNA region was used as a positive control. The number of copies in the main stock dilution was calculated as 2.4 × 10⁸, and nine serial 10-fold dilutions were prepared. Sterile distilled water was used as a negative control

The various concentrations of the extract (5, 2.5, and 1 µg/mL) were added to the *E. intestinalis*-infected cells and incubated at 37 °C in 5% CO_2_. The medium was replaced every 48 h, and a total of five doses of extract were administered. After 10 days, spores began to appear in the culture medium, and the experiment was terminated (Fig. 2A). Culture medium and cells were collected, and spore load was calculated by real-time PCR. The 5 µg/ml concentration of the extract decreased the spore DNA load significantly (*P* < 0.05). However, neither the 1 µg/mL nor the 2.5 µg/mL extracts caused a significant decrease in spore DNA load (*P* > 0.05) (Fig. 2B).

### Anti-amoebic Activity

Trophozoites and cysts were seeded in 96-well plates and exposed to various concentrations of plant extract. The number of viable trophozoites and cysts was counted using trypan blue staining at 24, 48, and 72 h. Table 1 shows the amoebicidal effects of the extract on the cyst and trophozoite forms of *A. castellanii*.

**Fig. 3:**
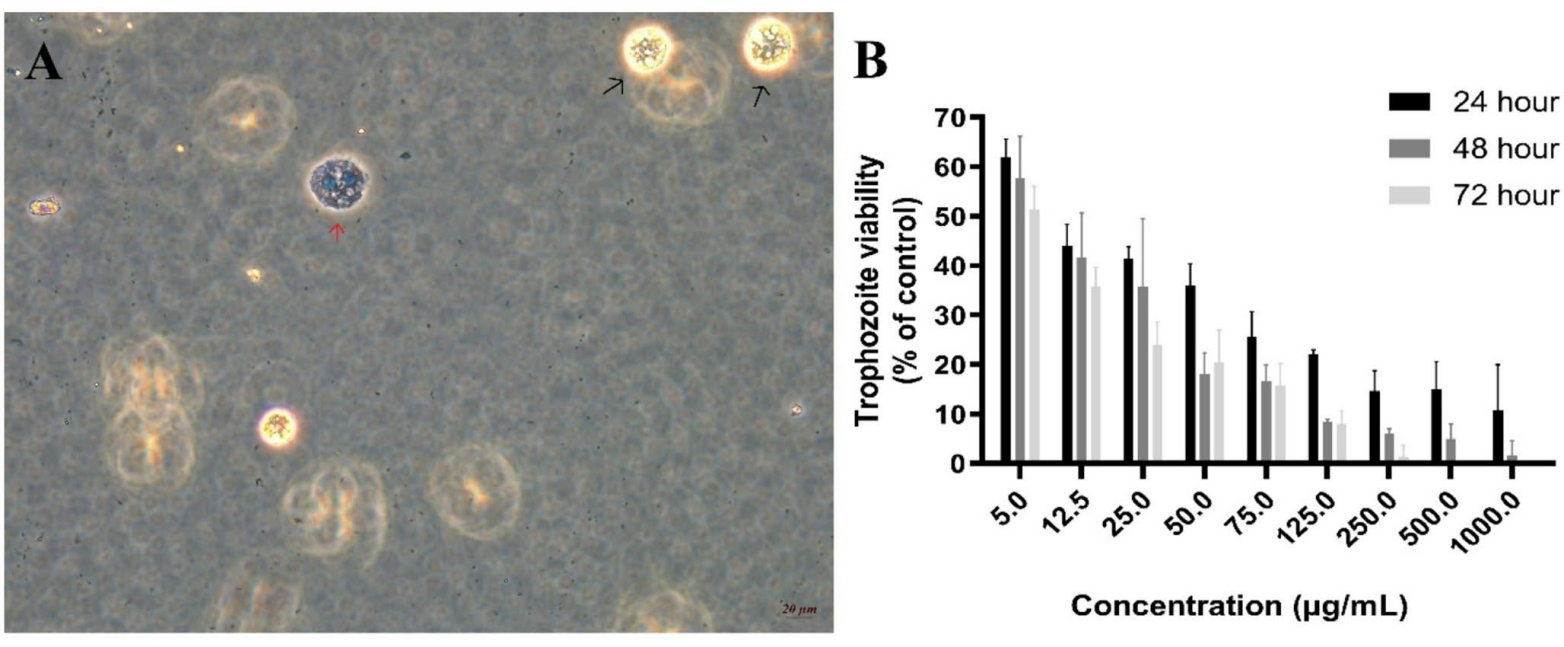
Amoebicidal effects of EA leaf extract on *Acanthamoeba castellanii* trophozoite forms. **A** Representative images of *(A) castellanii* viable (black arrows) and non-viable (red arrows) trophozoites. **B** Viability (%) of trophozoites treated with various concentrations of the extract (5, 12.5, 25, 50, 75, 125, 250, and 500 µg/mL), expressed relative to the untreated control group, which was set to 100% viability. Bars represent the mean ± SD of three independent experiments

Examining the effects of the plant extract on *A. castellanii* trophozoite forms (Fig. 3A) revealed that the viability rate of trophozoites decreased by 38% at a concentration of 5 µg/mL after 24 h. This decrease was statistically significant (*P* < 0.05). At this concentration, the viability was 58% and 52% after 48 and 72 h, respectively (Fig. 3B). A decrease in viability was observed over time at concentrations of 75 µg/mL and below, but this was not statistically significant (*P* > 0.05). However, a time-dependent decrease in viability was observed at concentrations of 125 µg/mL and above (*P* < 0.05) (Table 1).

**Fig. 4:**
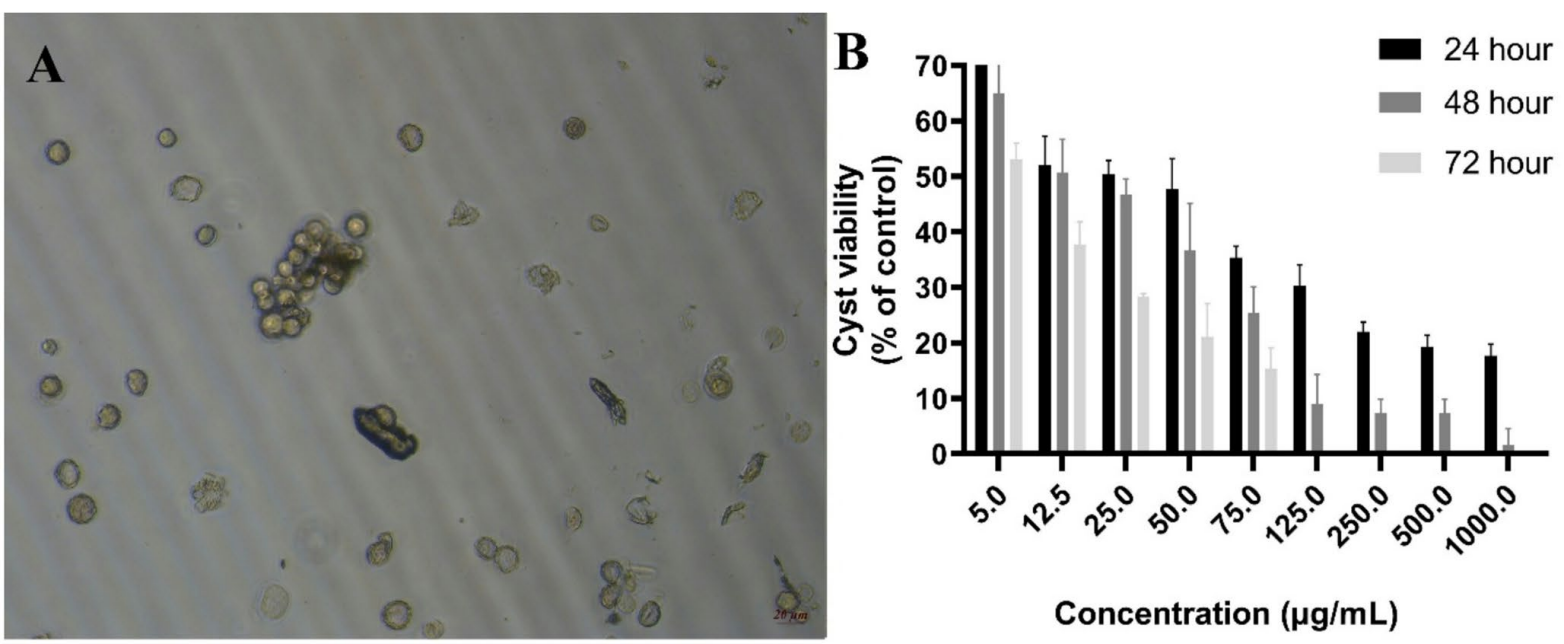
Amoebicidal effects of EA leaf extract on *Acanthamoeba castellanii* cyst forms. **A** Representative images of *A. castellanii* viable and non-viable cyst. **B** Viability (%) of trophozoites treated with various concentrations of the extract (5, 12.5, 25, 50, 75, 125, 250, and 500 µg/mL), expressed relative to the untreated control group, which was set to 100% viability. Bars represent the mean ± SD of three independent experiments

Examining the effects of the plant extract on *A. castellanii* cyst forms (Fig. 4A) revealed that the viability rate of cysts decreased by 30% at a concentration of 5 µg/mL after 24 h. This decrease was statistically significant. At this concentration, the viability was 65% and 53% after 48 and 72 h, respectively (Fig. 4B) (*P* < 0.05). In contrast to trophozoites, cysts exhibited a time-dependent decrease in viability even at a concentration of 5 µg/mL, and a similar trend was observed at all concentrations (Table 1) (*P* < 0.05).

### Anti-leishmanial Activity

**Fig. 5:**
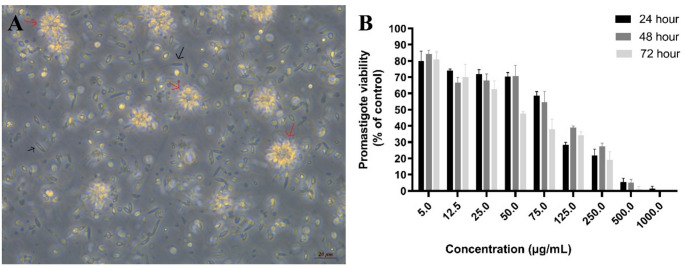
Anti-leishmanial effects of EA leaf extract on *Leishmania major* promastigotes. **A** Representative images of promastigote morphology. Promastigotes (black arrows) and rosette structures formed by promastigotes (red arrows). **B** Viability (%) of promastigotes treated with various concentrations of the extract (5, 12.5, 25, 50, 75, 125, 250, 500, and 1000 µg/mL), expressed relative to the untreated control group, which was set to 100% viability. Bars represent the mean ± SD of three independent experiments

Promastigotes (Fig. 5A) were seeded in 96-well plates and exposed to various concentrations of plant extracts. The number of viable promastigotes was counted at 24, 48, and 72 h. Table 1 shows the anti-leishmanial effects of the extract on the cyst and trophozoite forms of *L. major*.

Examining the effects of the plant extract on *L. major* promastigote forms revealed that promastigote viability decreased by approximately 20% at a concentration of 5 µg/mL after 24 h (*P* < 0.05). However, at the same concentration, viability values of 84% and 81% were observed at 48 and 72 h, respectively, indicating no further time-dependent reduction (Fig. 5B). Similarly, at concentrations of 25 µg/mL and below, no statistically significant differences in viability were observed among the 24, 48, and 72 h time points (*P* > 0.05). In contrast, a significant time-dependent decrease in viability was detected at higher concentrations (50, 75, and 125 µg/mL) (*P* < 0.05) (Table 1).

**Table 1:** The amoebicidal and anti-leishmanial effects of the extract

	Time (hour)	Concentrations (µg/mL)								
		5	12.5	25	50	75	125	250	500	1000
Trophozoite	24	62 ± 4	44 ± 4	41 ± 3	36 ± 4	26 ± 5	22 ± 1^a^	15 ± 4^bc^	15 ± 6^de^	11 ± 9
	48	58 ± 9	42 ± 9	36 ± 14	18 ± 4	17 ± 3	8 ± 1	6 ± 1^b^	5 ± 3^d^	2 ± 3
	72	52 ± 5	36 ± 4	24 ± 5	20 ± 6	16 ± 4	8 ± 2^a^	1 ± 3^c^	0 ± 0^e^	0 ± 0
Cyst	24	70 ± 8^a^	52 ± 5^b^	50 ± 2^cd^	47 ± 6^e^	35 ± 2^fg^	31±4^ıj^	22 ± 2^kl^	19 ± 2^no^	17 ± 2^yz^
	48	65 ± 6	51 ± 6	47 ± 3^c^	37 ± 8	25 ± 5^fh^	9±5^ı^	7 ± 3^km^	7 ± 3^nv^	2 ± 3^yx^
	72	53 ± 3^a^	30 ± 8^b^	19 ± 3^d^	13 ± 3^e^	9 ± 8^gh^	0 ± 0^j^	0 ± 0^lm^	0 ± 0^ov^	0 ± 0^zx^
Promastigote	24	80 ± 6	74 ± 1	72 ± 3	70 ± 3^ab^	59 ± 2^cd^	28 ± 1^ef^	22 ± 4	5 ± 3	1 ± 1
	48	84 ± 2	67 ± 3	68 ± 4	71 ± 7^a^	55 ± 7^c^	39 ± 1^eg^	27 ± 2	5 ± 2	0 ± 0
	72	81 ± 5	70 ± 8	63 ± 5	48 ± 1^b^	38 ± 7^d^	34 ± 2^fg^	19 ± 5	1 ± 2	0 ± 0

The viability was calculated based on the results of the control group.Data were expressed as mean ± SD; differences between columns marked with different letters were statistically significant (*p* < 0.05)

## Discussion

Parasitic diseases are a major global health concern. Treatment of these diseases presents many challenges, including drug resistance, limited efficacy, and the recurrence of infection. This necessitates the development of new treatment options [29–31]. EA is known for its anti-oxidant, anti-inflammatory, anti-microbial, and anti-cancer properties, and various parts of the plant have been used as traditional treatments for centuries [13, 14]. However, not a single study has shown the effect of EA leaf extract on parasites. This study comprehensively evaluated the in vitro effects of the ethanol extract of EA leaves on *E. intestinalis*, *A. castellanii*, and *L. major*. The results indicate that EA exhibits an anti-parasitic effect on these three parasites.

The DPPH radical scavenging capacity, total phenolic, and total flavonoid content of the extracts were found to be 157.47 mg GAE/g, 32.34 mg RE/g, and 279.32 mg TE/g, respectively. These findings reflect higher levels than those reported in the literature. For instance, one study reported a phenolic content of 8–10 mg GAE/100 g FW in the methanol extract of EA leaves. Similarly, flavonoid content was reported as quite low in that study (3–6 mg QE/100 g FW) [11]. This significant variation in content may be attributed to factors such as the geographical location of sample collection, the timing of leaf collection, the drying method used, and the extraction protocol. Our findings suggest that EA leaves are effective anti-oxidants with considerable potential for biological activity studies.

Microsporidia are obligate intracellular pathogens that can infect many vertebrates and invertebrates, causing significant economic losses [32]. This diverse group comprises over 200 genera and 1200 species, and is known to cause fatal infections, particularly in immunocompromised individuals. *E. intestinalis* is one of the most common microsporidia in humans [33]. Albendazole, a benzimidazole derivative with broad-spectrum anthelmintic and anti-fungal properties, is used to treat this species. Although albendazole is frequently used to treat microsporidiosis, it is sometimes only partially effective, and recurrences have been reported [31]. Recent studies suggest that herbal compounds may be highly effective against microsporidian infections. In an in vivo study of the effects of various plant extracts on *Nosema ceranae* (the causative agent of choline mortality in honeybees), Porrini et al. proposed the use of natural substances as an alternative to anti-parasitic treatment [34]. An in vitro study on *E. intestinalis* found that thymoquinone (a derivative of *Nigella sativa*) reduced spore density [35]. Furthermore, natural products such as propolis and royal jelly have been reported to inhibit the same parasite [36]. This study also found that a non-cytotoxic concentration of EA leaves extract inhibited parasite growth. Taken together, these findings suggest that herbal and natural products show promise in the management of microsporidian infections.

Cutaneous leishmaniasis (CL) is a neglected tropical disease caused by protozoan parasites of the genus Leishmania transmitted by female phlebotomine sandflies [37]. It is estimated that 600,000 to 1 million new cases occur worldwide each year. Various drugs are available to treat CL. However, most of these drugs used to treat Leishmaniasis have many limitations, such as low sensitivity, toxicity, high cost, and drug resistance [37, 38].

The effects of herbal extracts on leishmaniasis have been investigated in numerous studies. Namaei et al. reported that *Prosopis farcta* fruit and leaf extracts exhibited significant inhibitory activity against both amastigotes and promastigotes, with IC₅₀ values of 0.9 and 1.1 mg/ml, respectively. It was also emphasized that there was a 97.3% reduction in parasite load in infected macrophages treated using fruit extract [39]. Similarly, *Zingiber officinale* (ginger) extract has been shown to exhibit significant in vitro anti-leishmanial activity against *L. major*, with IC₅₀ values of approximately 56 µg/ml and 75 µg/ml for promastigotes and amastigotes, respectively, after 72 h of incubation [40]. *Urtica dioica* (nettle) extract has been shown to inhibit parasites directly and enhance the immune response of host cells. It reduces parasite load in infected macrophages by increasing nitric oxide (NO) and proinflammatory cytokine production [41]. Another study showed that *Calotropis procera* extract suppresses the proliferation of *L. major* promastigotes and exhibits effects that enhance oxidative stress and the Th1 response [42]. Taken together, these findings suggest that different plant-derived metabolites have the potential to control *L. major* infections through direct anti-parasitic effects and immunomodulation. The EA extract used in our study was also found to significantly anti-parasitic effect. Acanthamoeba species can cause serious infections, including acanthamoeba keratitis (AK) and granulomatous amoebic encephalitis (GAE) [43]. These free-living amoebas are commonly found in soil, freshwater, and the air [3, 44]. Currently, there is no FDA-approved standard treatment protocol for *Acanthamoeba* spp., and treatment typically involves long-term use of various topical anti-septics and anti-microbials. However, therapeutic success is limited, and relapses occur [45]. In recent years, the therapeutic potential of herbal extracts against Acanthamoeba species has been the subject of intensive research, with extracts from many different plants reported to exhibit significant amebicidal activity against both trophozoites and cysts. In particular, methanol, ethanol, or essential oil extracts of medicinal plants, such as *Thymus capitatus* [46], *Origanum* spp [47]., *Nigella sativa* [48], and *Allium sativum* [49], have been shown to exhibit strong anti-amebic effects in *A. castellanii*. Additionally, some studies have reported the anti-amoebic effects of certain plant-based secondary compounds, including betulinic acid, betulin, and vanillic acid. When tested against the cyst stage, these compounds were found to inhibit the encystation processes [50]. When the amoebicidal effect of the plant extract used in this study was evaluated, it was found that the lowest concentration reduced trophozoite viability by around 40% after 24 h and by almost 50% after 72 h. Similar results were observed for the cyst form. While cyst viability decreased by 30% at 24 h, this rate approached 50% at 72 h. As the Acanthamoeba cyst form is highly resistant to environmental stresses and existing treatments due to its dense [51], double-walled structure, a plant extract that is at least as effective against cysts as it is against trophozoites would provide a significant therapeutic advantage. An extract capable of targeting cysts reduces the risk of recurrence of infection [52] and offers the potential for more durable and reliable treatment than agents that kill only trophozoites. Therefore, potent activity against cysts is a critical feature that establishes a herbal extract as a viable therapeutic candidate.

In vitro validation is an essential preliminary step in anti-parasitic studies involving plant extracts. These models enable the assessment of anti-parasitic activity, dose–response relationships, and host cell cytotoxicity under controlled conditions, before in vivo experimentation. This approach facilitates the design of appropriate experiments and reduces the need for animal models. However, the direct use of crude plant extracts may be limited by factors such as low bioavailability, limited stability, and potential non-specific effects [53]. Therefore, future studies should consider formulation-based approaches, such as extract standardisation, nanoencapsulation, or targeted delivery systems, to enhance the stability, safety, and therapeutic potential of EA-derived compounds before advancing to in vivo and clinical investigations [54].

## Conclusion

In conclusion, the biological activity of plant extract derived from EA leaves against three different parasites: *A. castellanii*, *E.intestinalis*, and *L. major*, suggests that these extract may be promising alternatives in the management of parasitic infections. The observed anti-parasitic effects may be partly attributed to the presence of bioactive phytochemicals. Previous phytochemical studies [55] have shown that EA contains a diverse range of bioactive constituents, including phenolic compounds, flavonoids and volatile secondary metabolites such as phytol, nonanal and Z-3-hexenyl benzoate. Plants rich in these classes of bioactive compounds have been reported in the literature to exhibit anti-parasitic activity [56–58]. However, the effects of the extract must be confirmed through comprehensive in vivo testing, and the cellular and molecular mechanisms underlying these anti-parasitic activities must be elucidated.

## Data Availability

No datasets were generated or analysed during the current study.
